# The genome sequence of the cave orb-weaver, *Meta bourneti* (Simon, 1922)

**DOI:** 10.12688/wellcomeopenres.18638.1

**Published:** 2022-12-22

**Authors:** Sergio Henriques, Olga Sivell, Natural History Museum Genome Acquisition Lab, Darwin Tree of Life Barcoding collective, Wellcome Sanger Institute Tree of Life programme, Wellcome Sanger Institute Scientific Operations: DNA Pipelines collective, Tree of Life Core Informatics collective, Darwin Tree of Life Consortium

**Affiliations:** 1 Indianapolis Zoo, Indiana, USA; 2 Department of Life Sciences, Natural History Museum, London, UK

**Keywords:** Meta bourneti, cave spider, genome sequence, chromosomal, Arachnida

## Abstract

We present a genome assembly from an individual male *Meta bourneti* (the cave orb-weaver; Arthropoda; Arachnida; Araneae; Tetragnathidae). The genome sequence is 1,383 megabases in span. Most of the assembly is scaffolded into 13 chromosomal pseudomolecules, including half coverage of two X sex chromosomes. The mitochondrial genome has also been assembled and is 15.8 kilobases long.

## Species taxonomy

Eukaryota; Metazoa; Ecdysozoa; Arthropoda; Chelicerata; Arachnida; Araneae; Araneomorphae; Entelegynae; Araneoidea; Tetragnathidae; *Meta*; *Meta bourneti* (Simon, 1922) (NCBI txid:1926196).

## Background

Cave orb-weavers ( *Meta* spp.) are large (10–16 mm), glossy, dark brown spiders with long legs. They belong to the Tetragnathidae family of long-jawed orb-weaving spiders ( Bee *et al.*, 2020). *Meta bourneti* closely resembles the more common and widespread *Meta menardi* (Latreille, 1804), and examination of the epigyne (female) or male palpal organs is required for a positive identification ( Roberts, 1995).

In Britain, *M. bourneti* occurs mainly in southern and eastern England, with a wide but scattered distribution. The species is nationally scarce ( Bee *et al.,* 2020). It was first found in Britain in 1941 ( Browning & Tams, 1944), but may have been previously overlooked. Some data indicate it is invasive ( Hesselberg, 2021; Mammola, 2017). *M. bourneti* requires damp and dark conditions. In southern Europe it is found in subterranean sites such as caves and mines, while in Britain it prefers above-ground sites like hollow trees and synanthropic habitats such as telephone junction boxes, manhole covers, culverts, sewers and drains, icehouses, bunkers and air-raid shelters, burial vaults, and compost bins ( Bee *et al.,* 2020; Collyer, 2013; Cooke, 1981; Cropper, 1997; Halsted, 2000; Henriques, 2021; Hesselberg, 2021; Milner, 2013; Pendleton & Pendleton, 2009; Prince, 2018; Twissel, 2019). British caves and mines are more commonly occupied by the native *M. menardi* ( Hesselberg, 2021).

*M. bourneti* produces a rudimentary orb web with a reduced number of frame threads and radii attaching directly to cave walls. These webs capture flying as well as non-flying prey ( Hesselberg *et al.,* 2019). Courtship occurs after a female catches a prey item. The male approaches the female and mates with her while she is actively feeding on her catch. The egg sacs are round, relatively large and attached near the web, suspended on silk threads ( Roberts, 1995). *Meta* species have high dispersal ability during the epigean (ground-dwelling) ecophase in their life cycle. The spiderlings switch from negative to positive phototaxis, exit the cave and are carried away by wind (ballooning). They spend part of their juvenile life (second to third instar) outside ( Mammola & Isaia, 2014). Young *Meta* spiders have a strong black-and-white body pattern and annulated legs. They darken abruptly at the moult from the third to fourth instar and lose most of the markings, giving them more adult-like appearance ( Pennington, 1979). At this stage they also switch to negative phototaxis and proceed to colonise dark, humid sites such as caves, drains, vaults, and so on. This hypogean (subterranean) ecophase lasts for the remainder of their life cycle ( Mammola & Isaia, 2014).

The high-quality genome sequence described here is the first one reported for *M. bourneti* and has been generated as part of the Darwin Tree of Life project. It will aid in understanding the biology and ecology of the species.

## Genome sequence report

The genome was sequenced from one male *M. bourneti* (qqMetBour1) collected from Highgate Cemetery, London, UK, (51.568, –0.149). A total of 25-fold coverage in Pacific Biosciences single-molecule HiFi long reads and 48-fold coverage in 10X Genomics read clouds were generated. Primary assembly contigs were scaffolded with chromosome conformation Arima2 Hi-C data. Manual assembly curation corrected 276 missing/misjoins, reducing the scaffold number by 29.85%, and increasing the scaffold N50 by 4.31%. The final assembly has a total length of 1,383 Mb in 470 sequence scaffolds with a scaffold N50 of 104.1 Mb ( Table 1). Most (98.63%) of the assembly sequence was assigned to 13 chromosomal-level scaffolds, representing 11 autosomes and the X1 and X2 sex chromosomes ( Figure 3– Figure 6; Table 2). The two X chromosomes identified in this assembly had half coverage ( Figure 6), leading to the denoting of this individual as male, as female spiders are XX and would therefore have diploid coverage of the X chromosomes. Chromosome-scale scaffolds confirmed by the Hi-C data are named in order of size. The assembly has a BUSCO v5.3.2 ( Manni *et al.*, 2021) completeness of 97.2% (single 91.6%, duplicated 5.6%) using the arachnida_odb10 reference set. While not fully phased, the assembly deposited is of one haplotype. Contigs corresponding to the second haplotype have also been deposited.

**Table 1.** Genome data for *M. bourneti*, qqMetBour1.1.

Project accession data	
Assembly identifier	qqMetBour1.1
Species	*Meta bourneti*
Specimen	qqMetBour1
NCBI taxonomy ID	1926196
BioProject	PRJEB48587
BioSample ID	SAMEA9066041
Isolate information	Male, whole organism, cephalothorax (HiC)
Assembly metrics *	
Base pair QV	57.1 (Benchmark: ≥ 50)
*k*-mer completeness	99.99% (Benchmark: ≥ 95%)
BUSCO **	C:97.2%[S:91.6%,D:5.6%],F:0.8%,M:1.9%,n:2934 (Benchmark: C ≥ 95%)
Percentage of assembly mapped to chromosomes	98.63% (Benchmark: ≥ 95%)
Sex chromosomes	X1 and X2 (Benchmark: localised homologous pairs)
Organelles	Mitochondrion (Benchmark: complete single alleles)
Raw data accessions	
PacificBiosciences SEQUEL II	ERR7254654, ERR7254655
10X Genomics Illumina	ERR7253235–ERR7253238
Hi-C Illumina	ERR7253239
Genome assembly	
Assembly accession	GCA_933210815.1
*Accession of alternate haplotype*	GCA_933210875.1
Span (Mb)	1,383
Number of contigs	944
Contig N50 length (Mb)	8.6
Number of scaffolds	470
Scaffold N50 length (Mb)	104.1
Longest scaffold (Mb)	120.0

* Assembly metric benchmarks are adapted from column VGP-2020 of “Table 1: Proposed standards and metrics for defining genome assembly quality” from ( Rhie *et al.*, 2021). ** BUSCO scores based on the arachnida_odb10 BUSCO set using v5.3.2. C = complete [S = single copy, D = duplicated], F = fragmented, M = missing, n = number of orthologues in comparison. A full set of BUSCO scores is available at https://blobtoolkit.genomehubs.org/view/qqMetBour1.1/dataset/CAKOFA01/busco.

**Table 2.** Chromosomal pseudomolecules in the genome assembly of *M. bourneti*, qqMetBour1.

INSDC accession	Chromosome	Size (Mb)	GC%
OW119922.1	1	119.99	35.3
OW119933.1	X1	108.23	35.2
OW119923.1	2	118.36	35.3
OW119924.1	3	114.77	35.2
OW119934.1	X2	103.99	35
OW119925.1	4	110.92	35.6
OW119926.1	5	104.82	35.6
OW119927.1	6	104.07	35.5
OW119928.1	7	100.56	35.7
OW119929.1	8	99.47	35.6
OW119930.1	9	96.52	36.2
OW119931.1	10	92.22	36.5
OW119932.1	11	89.97	36.7
OW119935.1	MT	0.02	30.2

## Methods

### Sample acquisition and nucleic acid extraction

**Figure 1.**
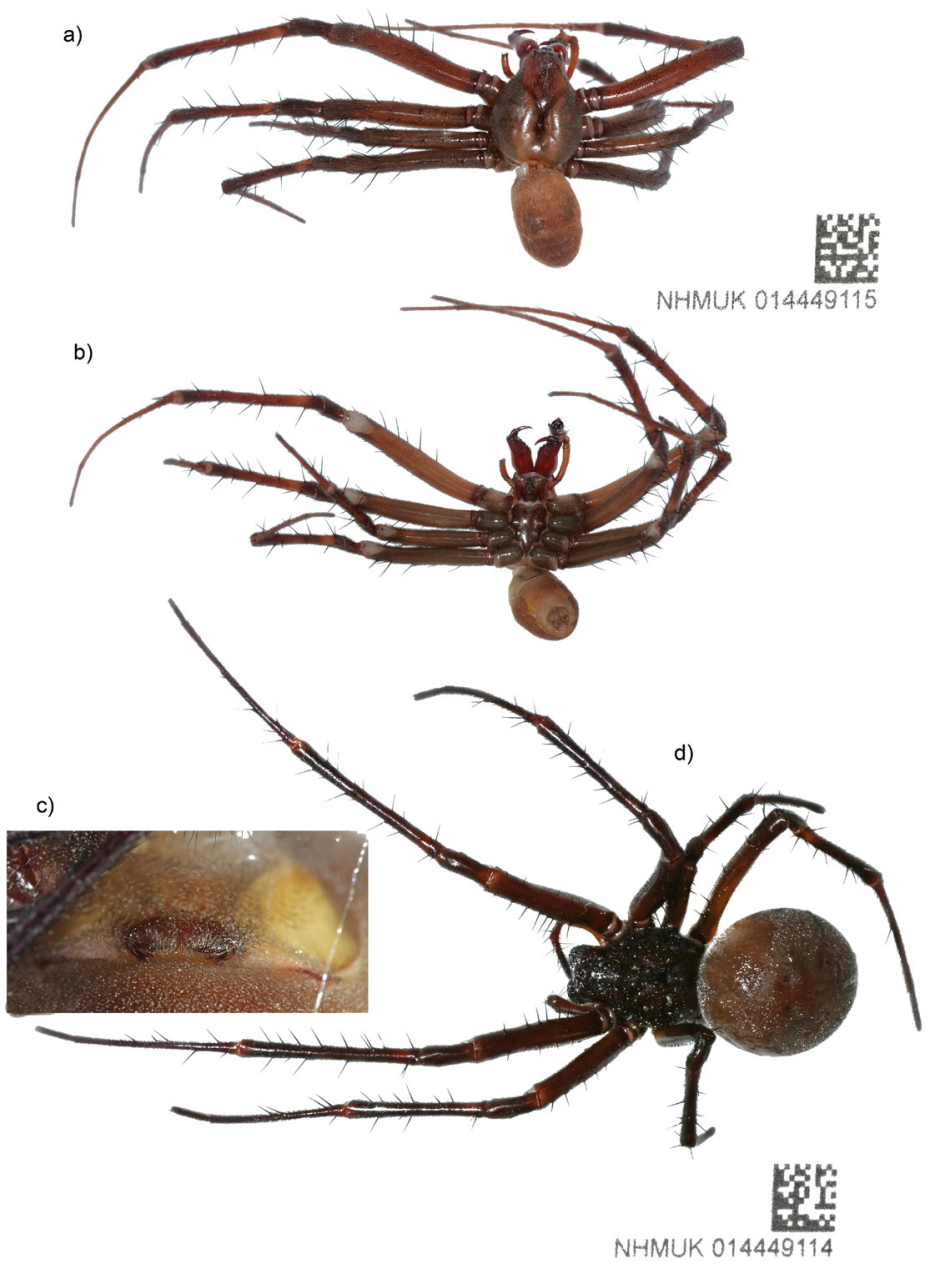
Image of the *M. bourneti* (qqMetBour1) specimens used for genome sequencing and as a voucher. **a**) Male habitus in dorsal view. **b**) Male habitus in ventral view. **c**) Female epigyne. **d**) Female habitus in dorsal view. Photographs by Olga Sivell and the Trustees of the Natural History Museum, London.

**Figure 2.**
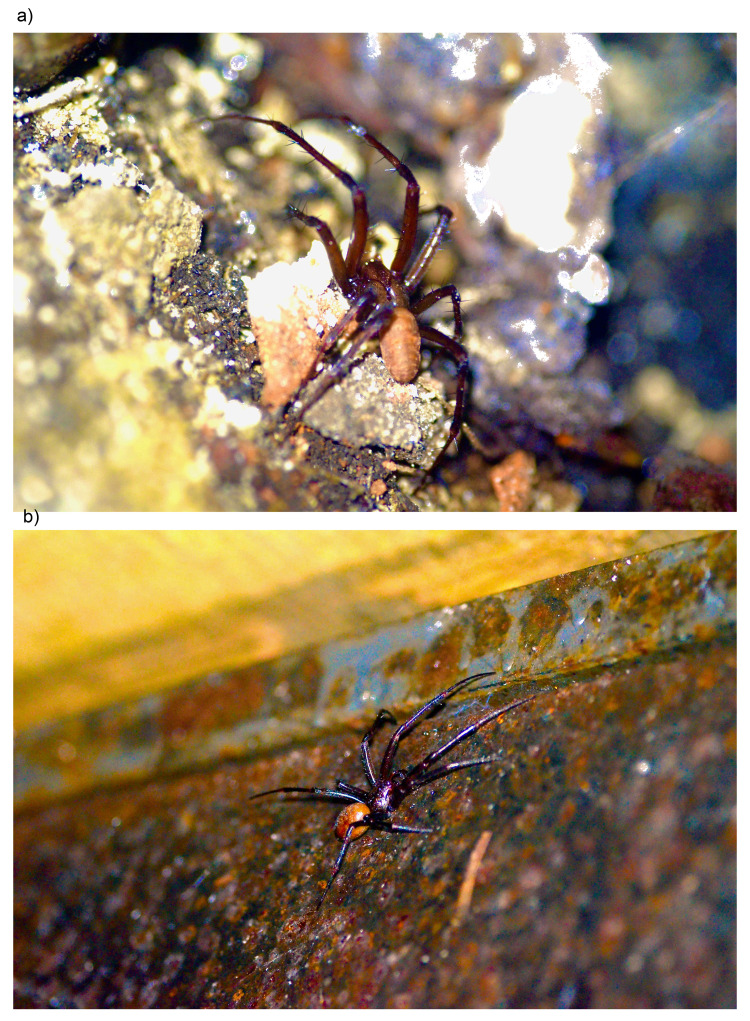
Live *M. bourneti* specimens at Highgate Cemetery, London: **a**) Live male specimen. **b**) Live female specimen. Photographs by Sergio Henriques.

A live male and female *M. bourneti* ( Figure 1 and Figure 2) were collected by hand from a vault in the Egyptian Avenue at Highgate Cemetery (51.568, –0.149), TQ283870, London, England, and identified by Sergio Henriques, Indianapolis Zoo, US, following Roberts (1995). The female specimen ( Figure 1c) and d)) was preserved in ethanol as a voucher (NHMUK014449114) and a sample from its leg was submitted for COI barcoding. The male specimen ( Figure 1a) and b)) was snap-frozen on dry ice. The tissue samples taken from it were stored in a CoolRack prior to nucleic acid extraction. A pedipalp was preserved in ethanol to serve as a morphological voucher (NHMUK014449115). The vouchers were deposited at the Natural History Museum in London.

**Figure 3.**
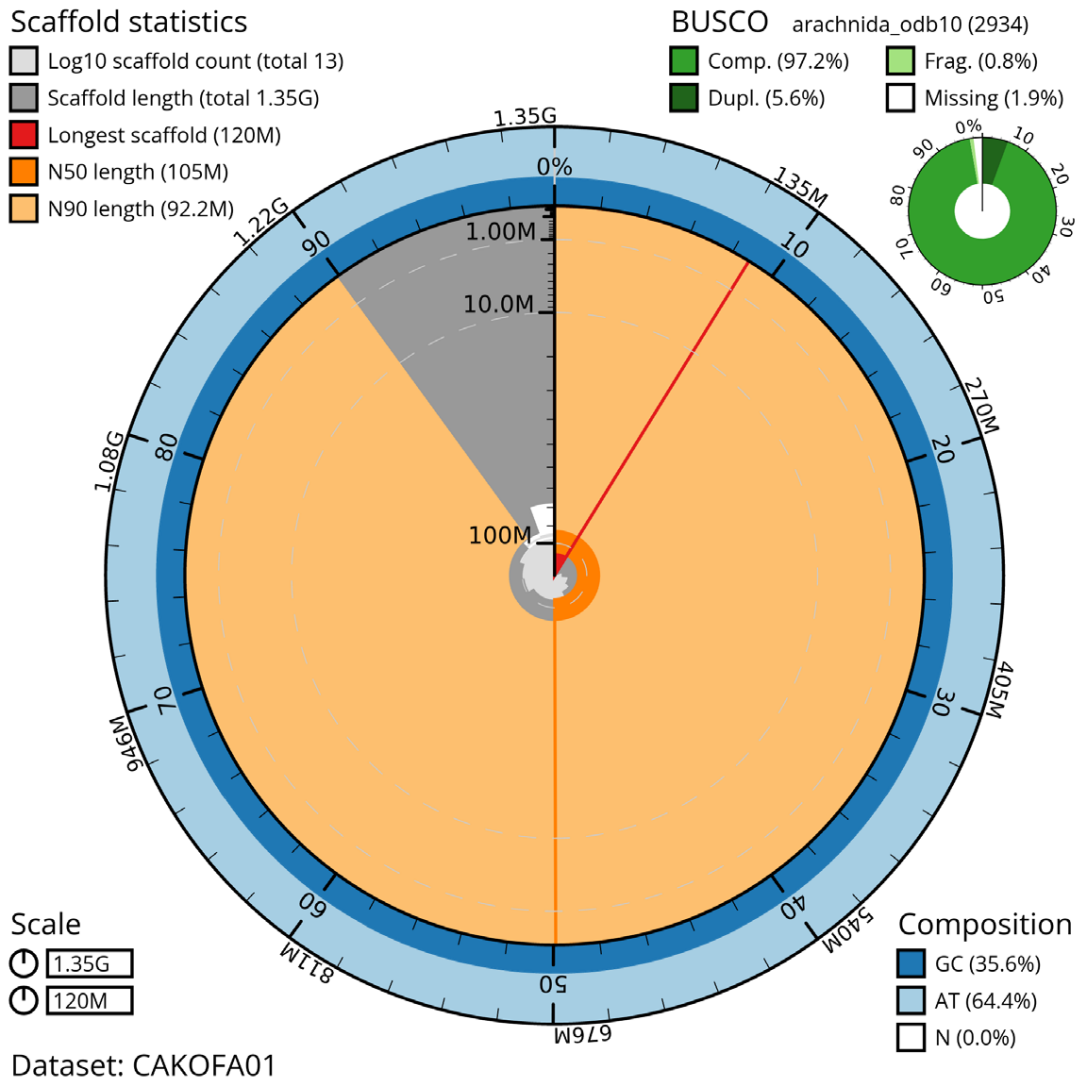
Genome assembly of *M. bourneti* qqMetBour1.1: metrics. The BlobToolKit Snailplot shows N50 metrics and BUSCO gene completeness. The main plot is divided into 1,000 size-ordered bins around the circumference with each bin representing 0.1% of the 1,351,064,048 bp assembly. The distribution of chromosome lengths is shown in dark grey with the plot radius scaled to the longest chromosome present in the assembly (119,993,891 bp, shown in red). Orange and pale-orange arcs show the N50 and N90 chromosome lengths (104,822,047 and 92,215,385 bp), respectively. The pale grey spiral shows the cumulative chromosome count on a log scale with white scale lines showing successive orders of magnitude. The blue and pale-blue area around the outside of the plot shows the distribution of GC, AT and N percentages in the same bins as the inner plot. A summary of complete, fragmented, duplicated and missing BUSCO genes in the arachnida_odb10 set is shown in the top right. An interactive version of this figure is available at https://blobtoolkit.genomehubs.org/view/qqMetBour1.1/dataset/CAKOFA01/snail.

**Figure 4.**
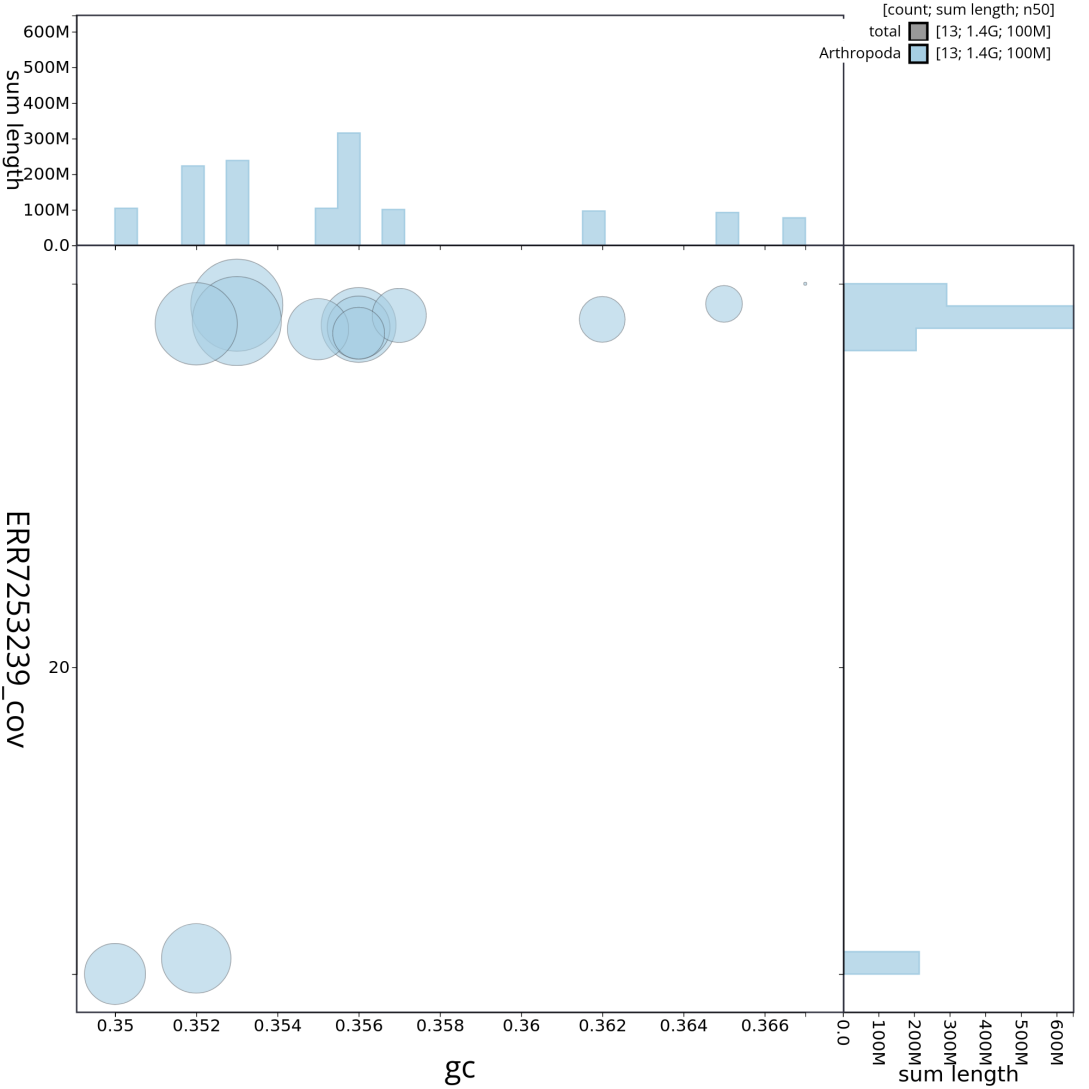
Genome assembly of *M. bourneti* qqMetBour1.1: GC coverage. BlobToolKit GC-coverage plot. Chromosomes are coloured by phylum. Circles are sized in proportion to chromosome length. Histograms show the distribution of chromosome length sum along each axis. An interactive version of this figure is available at https://blobtoolkit.genomehubs.org/view/qqMetBour1.1/dataset/CAKOFA01/blob.

**Figure 5.**
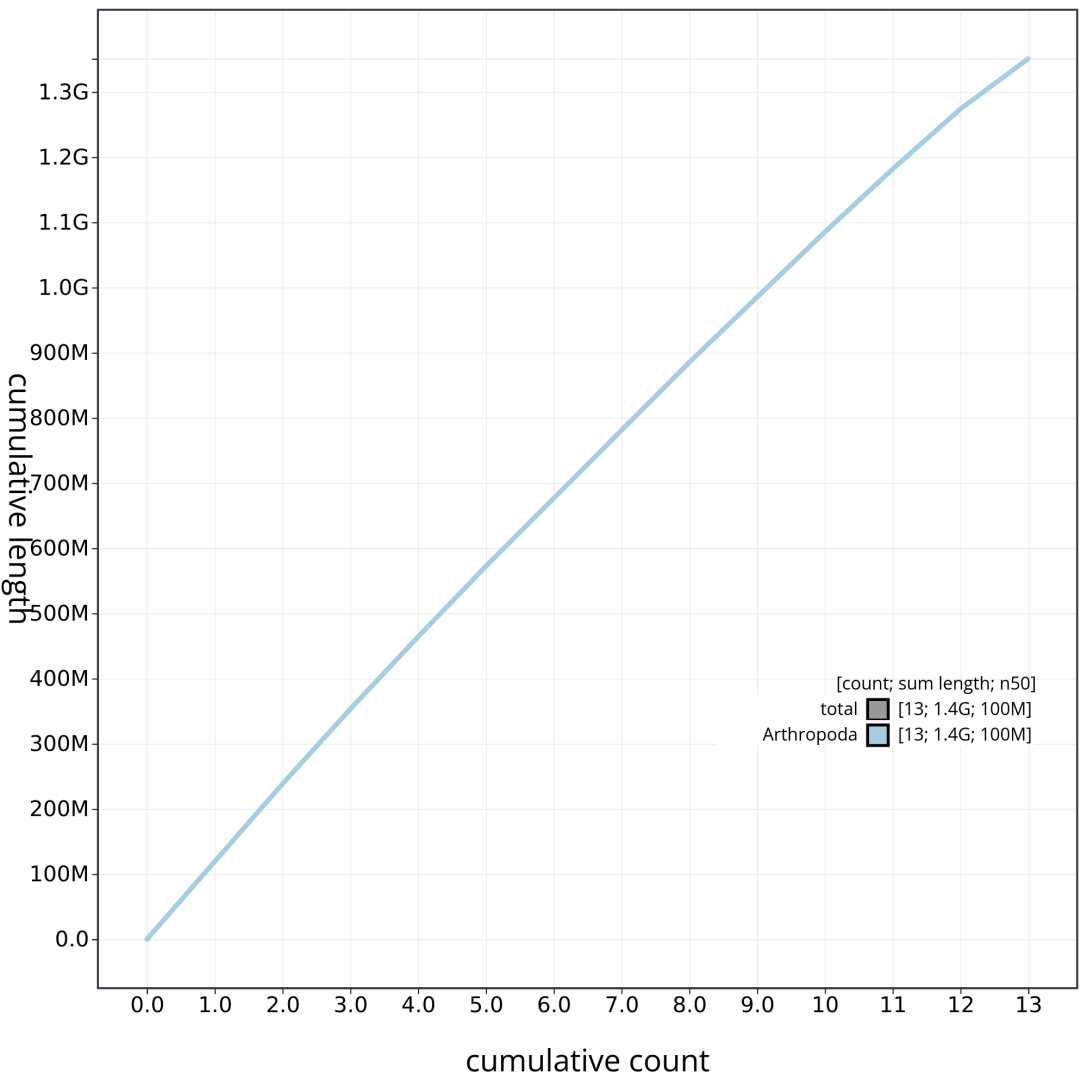
Genome assembly of *M. bourneti* qqMetBour1.1: cumulative sequence. BlobToolKit cumulative sequence plot. The grey line shows cumulative length for all chromosomes. Coloured lines show cumulative lengths of chromosomes assigned to each phylum using the buscogenes taxrule. An interactive version of this figure is available at https://blobtoolkit.genomehubs.org/view/qqMetBour1.1/dataset/CAKOFA01/cumulative.

**Figure 6.**
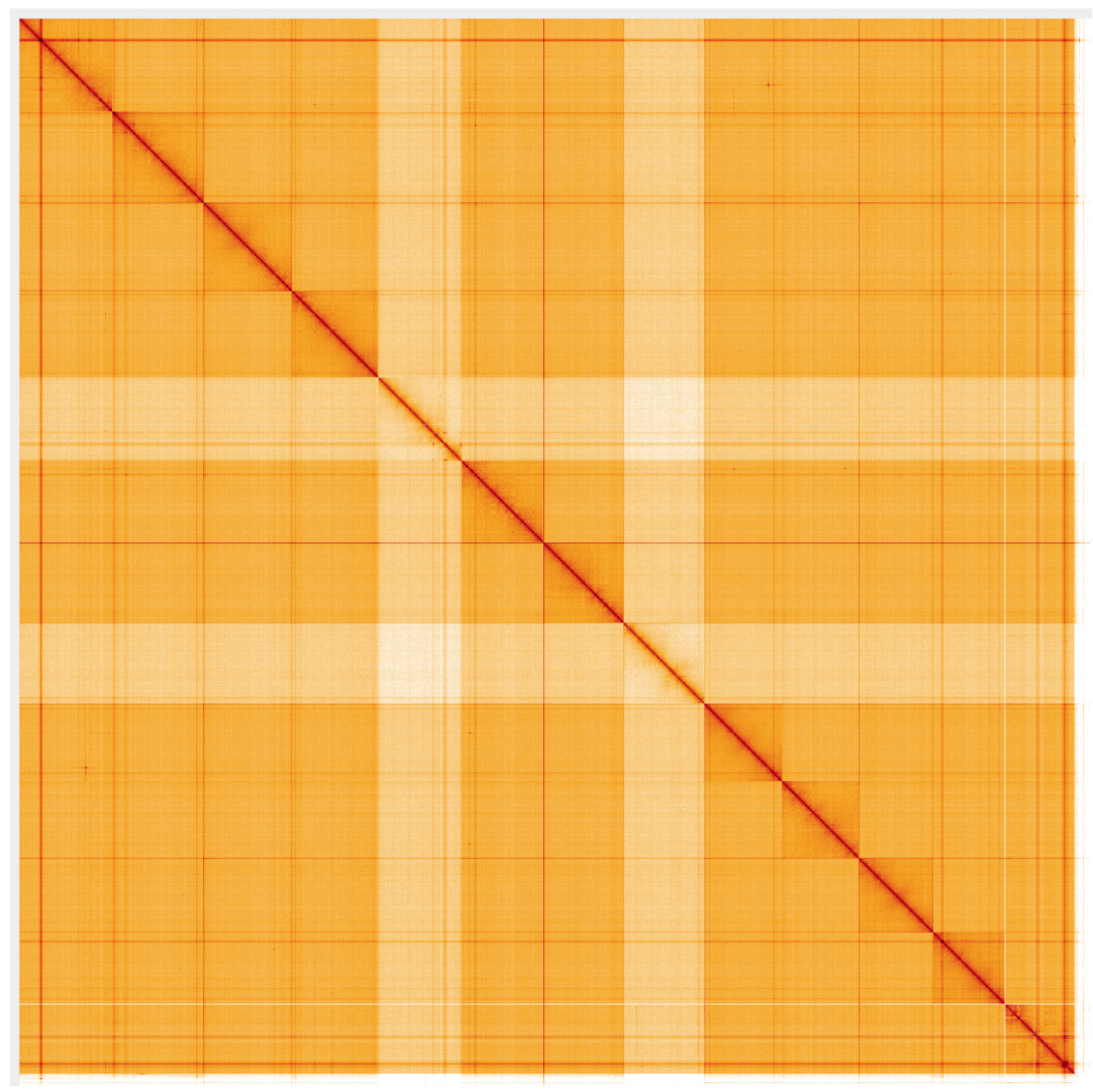
Genome assembly of *M. bourneti* qqMetBour1.1: Hi-C contact map. Hi-C contact map of the qqMetBour1.1 assembly, visualised using HiGlass. Chromosomes are shown in order of size from left to right and top to bottom. An interactive version of this figure may be viewed at https://genome-note-higlass.tol.sanger.ac.uk/l/?d=OWXvCTFdQe28ZreLawJYyw

DNA was extracted at the Tree of Life laboratory, Wellcome Sanger Institute. The qqMetBour1 sample was weighed and dissected on dry ice with tissue set aside for Hi-C sequencing. Tissue was cryogenically disrupted to a fine powder using a Covaris cryoPREP Automated Dry Pulveriser, receiving multiple impacts. High molecular weight (HMW) DNA was extracted using the Qiagen MagAttract HMW DNA extraction kit. Low molecular weight DNA was removed from a 20 ng aliquot of extracted DNA using 0.8X AMpure XP purification kit prior to 10X Chromium sequencing. A minimum of 50 ng DNA was submitted for 10X sequencing. HMW DNA was sheared into an average fragment size of 12–20 kb in a Megaruptor 3 system with speed setting 30. Sheared DNA was purified by solid-phase reversible immobilisation using AMPure PB beads with a 1.8X ratio of beads to sample to remove the shorter fragments and concentrate the DNA sample. The concentration of the sheared and purified DNA was assessed using a Nanodrop spectrophotometer and Qubit Fluorometer and Qubit dsDNA High Sensitivity Assay kit. Fragment size distribution was evaluated by running the sample on the FemtoPulse system.

### Sequencing

Pacific Biosciences HiFi circular consensus and 10X Genomics read cloud DNA sequencing libraries were constructed according to the manufacturers’ instructions. DNA sequencing was performed by the Scientific Operations core at the WSI on Pacific Biosciences SEQUEL II (HiFi) and Illumina NovaSeq 6000 (10X) instruments. Hi-C data were also generated from cephalothorax tissue of qqMetBour1 using the Arima v2 kit and sequenced on the Illumina NovaSeq 6000 instrument.

### Genome assembly

The assembly process for qqMetBour1.1 included the following sequence of steps: assembly was carried out with hifiasm ( Cheng *et al.*, 2021) and haplotypic duplication was identified and removed with purge_dups ( Guan *et al*., 2020). One round of polishing was performed by aligning 10X Genomics read data to the assembly with longranger align, calling variants with freebayes ( Garrison & Marth, 2012). The assembly was then scaffolded with Hi-C data ( Rao *et al.,* 2014) using YaHS ( Zhou *et al.,* 2022). The assembly was checked for contamination and corrected using the gEVAL system ( Chow *et al.*, 2016) as described previously ( Howe *et al.,* 2021). Manual curation ( Howe *et al.,* 2021) was performed using gEVAL, HiGlass ( Kerpedjiev *et al.*, 2018) and Pretext ( Harry, 2022). The mitochondrial genome was assembled using MitoHiFi ( Uliano-Silva *et al.*, 2021), which performed annotation using MitoFinder ( Allio *et al.*, 2020). The genome was analysed and BUSCO scores were generated within the BlobToolKit environment ( Challis *et al.*, 2020). Table 3 contains a list of all software tool versions used, where appropriate.

**Table 3.** Software tools and versions used.

Software tool	Version	Source
Hifiasm	0.15.3	( Cheng *et al.*, 2021)
purge_dups	1.2.3	( Guan *et al.*, 2020)
SALSA2	2.2	( Ghurye *et al.*, 2019)
longranger align	2.2.2	https://support.10xgenomics.com/genome-exome/ software/pipelines/latest/advanced/other-pipelines
freebayes	1.3.1-17-gaa2ace8	( Garrison & Marth, 2012)
MitoHiFi	2.0	( Uliano-Silva *et al.*, 2021)
gEVAL	N/A	( Chow *et al.*, 2016)
HiGlass	1.11.6	( Kerpedjiev *et al.*, 2018)
PretextView	0.2.x	( Harry, 2022)
BlobToolKit	3.3.10	( Challis *et al.*, 2020)
YaHS	1.0	( Zhou *et al.,* 2022)

## Data Availability

European Nucleotide Archive: *Meta bourneti* Accession number PRJEB48587; https://identifiers.org/ena.embl/PRJEB48587 ( Wellcome Sanger Institute, 2022). The genome sequence is released openly for reuse. The *Meta bourneti* genome sequencing initiative is part of the Darwin Tree of Life (DToL) project. All raw sequence data and the assembly have been deposited in INSDC databases. The genome will be annotated and presented through the Ensembl pipeline at the European Bioinformatics Institute. Raw data and assembly accession identifiers are reported in Table 1.

## Author information

Members of the Natural History Museum Genome Acquisition Lab are listed here: https://doi.org/10.5281/zenodo.4790042.

Members of the Darwin Tree of Life Barcoding collective are listed here: https://doi.org/10.5281/zenodo.4893703.

Members of the Wellcome Sanger Institute Tree of Life programme are listed here: https://doi.org/10.5281/zenodo.4783585.

Members of Wellcome Sanger Institute Scientific Operations: DNA Pipelines collective are listed here: https://doi.org/10.5281/zenodo.4790455.

Members of the Tree of Life Core Informatics collective are listed here: https://doi.org/10.5281/zenodo.5013541.

Members of the Darwin Tree of Life Consortium are listed here: https://doi.org/10.5281/zenodo.4783558.
