# Supplementary material for: The genome sequence of the cave orb-weaver, Meta bourneti (Simon, 1922)
Source: Wellcome Open Res. 2022 Dec 22;7:311. [Version 1] doi: 10.12688/wellcomeopenres.18638.1 (PMC9975426; doi:10.12688/wellcomeopenres.18638.1)
Supplement: Peer review history

# Peer review: Reviewer response for version 1

**DOI:** 10.21956/wellcomeopenres.20667.r54213

**Published:** 2023-02-06

**Authors:** Zhi-Sheng Zhang

**Affiliations:** 1 Key Laboratory of Eco-environments in Three Gorges Reservoir Region (Ministry of Education), School of Life Sciences, Southwest University, Chongqing, China

This manuscript presents a genome assembly for a tetragnathidae spider *Meta bourneti*. The genome sequence was assembled to chromosome level, including two X sex chromosomes. Also, the mitochondrial genome has also been assembled. The assembly has a BUSCO completeness of 97.2%. I am convinced that the assembly of *M. bourneti *is done well.

 I support the approved publication of this work.

Are sufficient details of methods and materials provided to allow replication by others?

Yes

Is the rationale for creating the dataset(s) clearly described?

Yes

Are the datasets clearly presented in a useable and accessible format?

Yes

Are the protocols appropriate and is the work technically sound?

Yes

Reviewer Expertise:

spider systematics and evolution

I confirm that I have read this submission and believe that I have an appropriate level of expertise to confirm that it is of an acceptable scientific standard.

# Peer review: Reviewer response for version 1

**DOI:** 10.21956/wellcomeopenres.20667.r54211

**Published:** 2023-01-25

**Authors:** Dimitar Dimitrov

**Affiliations:** 1 Department of Natural History, University Museum of Bergen, University of Bergen, Bergen, Norway

The present data note provides a high quality genome assembly of the spider *Meta bourneti*. Despite their high diversity, spider genomes have been challenging to generate and there are just a few high quality genomes available (one of them of other tetragnathid spider from the genus Tetragnatha). Thus, having more and high quality genomes is important contribution given increasing interest in spider comparative genomics in recent years.

 The manuscript is well written and uses proper methodology which is well explained. In addition to the nuclear genome assembly the authors also provide an assembled mitochondrial genome of *M. bourneti*.

 In my opinion the article can be published as it is as I do not have any major criticism.

Are sufficient details of methods and materials provided to allow replication by others?

Yes

Is the rationale for creating the dataset(s) clearly described?

Yes

Are the datasets clearly presented in a useable and accessible format?

Yes

Are the protocols appropriate and is the work technically sound?

Yes

Reviewer Expertise:

spider systematics and evolution; biogeography; macroevolution

I confirm that I have read this submission and believe that I have an appropriate level of expertise to confirm that it is of an acceptable scientific standard.

# Peer review: Reviewer response for version 1

**DOI:** 10.21956/wellcomeopenres.20667.r53771

**Published:** 2023-01-18

**Authors:** Trine Bilde, Jesper Bechsgaard

**Affiliations:** 1 Department of Biology, Aarhus University, Aarhus, Denmark

This paper presents the genome assembly of the spider *Meta bourneti *(Simon, 1922), **generated from material from a single male.

 The assembly was generated from sequence data from Pacific Biosciences, and primary assembly contigs were scaffolded with chromosome conformation Arima2 Hi-C data.

 The final assembly has a total length of 1,383 Mb in 470 sequence scaffolds with most of the assembly sequence assigned to 13 chromosomal-level scaffolds, representing 11 autosomes and the X1 and X2 sex chromosomes. The two X chromosomes have half coverage, since spiders show a X0 sex determination system, with males having a single copy of the X. The mitochondrial genome was assembled using MitoHiFi

 The paper presents genomic resources that are important for further functional or comparative analyses in evolutionary research on spiders.

Are sufficient details of methods and materials provided to allow replication by others?

Yes

Is the rationale for creating the dataset(s) clearly described?

Yes

Are the datasets clearly presented in a useable and accessible format?

Yes

Are the protocols appropriate and is the work technically sound?

Yes

Reviewer Expertise:

Evolutionary biology

We confirm that we have read this submission and believe that we have an appropriate level of expertise to confirm that it is of an acceptable scientific standard.
